# Dietary Short-Term Fiber Interventions in Arthritis Patients Increase Systemic SCFA Levels and Regulate Inflammation

**DOI:** 10.3390/nu12103207

**Published:** 2020-10-20

**Authors:** Kerstin Dürholz, Jörg Hofmann, Aida Iljazovic, Julian Häger, Sébastien Lucas, Kerstin Sarter, Till Strowig, Holger Bang, Jürgen Rech, Georg Schett, Mario M. Zaiss

**Affiliations:** 1 Department of Internal Medicine 3-Rheumatology and Immunology, Friedrich-Alexander University (FAU) Erlangen-Nürnberg and Universitätsklinikum Erlangen, 91054 Erlangen, Germany; kerstin.duerholz@uk-erlangen.de (K.D.); julian.haeger@fau.de (J.H.); sebastien.lucas@uk-erlangen.de (S.L.); kerstin.sarter-zaiss@uk-erlangen.de (K.S.); juergen.rech@uk-erlangen.de (J.R.); georg.schett@uk-erlangen.de (G.S.); 2 Deutsches Zentrum für Immuntherapie (DZI), 91054 Erlangen, Germany; 3 Department of Biology, Division of Biochemistry, Friedrich-Alexander University (FAU), 91058 Erlangen, Germany; joerg.hofmann@fau.de; 4 Helmholtz Centre for Infection Research, 38124 Braunschweig, Germany; aida.iljazovic@helmholtz-hzi.de (A.I.); till.strowig@helmholtz-hzi.de (T.S.); 5 Melio.Care GmbH, 91080 Marloffstein, Germany; info@melio.care

**Keywords:** high-fiber diet (HFD), microbial metabolites, short chain fatty acids (SCFA)

## Abstract

Chronic inflammatory diseases are often initiated and guided by the release of proinflammatory mediators. Rheumatoid arthritis (RA) is caused by an imbalance between the pro- and anti-inflammatory mediators in the joints, thereby favoring chronic inflammation and joint damage. Here, we investigate if short-term high-fiber dietary intervention shifts this towards anti-inflammatory mediators. Healthy controls (*n* = 10) and RA patients (*n* = 29) under routine care received daily high-fiber bars for 15 or 30 days, respectively. Stool and sera were analyzed for pro- and anti-inflammatory mediators. A high-fiber dietary intervention resulted in increased anti-inflammatory short-chain fatty acids (SCFA), decreased proarthritic cytokine concentrations, along with a durable shift in the Firmicutes-to-Bacteroidetes ratio. Together, these results further strengthen high-fiber dietary interventions as a practical approach complementing existing pharmacological therapies.

## 1. Introduction

Rheumatoid arthritis (RA) is a chronic inflammatory disorder with an autoimmune etiopathogenesis. It affects about 1% of the population worldwide, and is characterized by chronic inflammation of the synovium, particularly of small joints, which often leads to cartilage and bone destruction [1]. A large number of proinflammatory mediators, such as cytokines, are involved in the pathogenesis of RA [2]. It is now clear that inflammation, articular destruction, and the comorbidities associated with RA are mainly triggered by cytokines, ensuring the success of current RA treatment strategies of blocking proinflammatory cytokines such as TNFα, IL-1β or IL-6. However, the cytokine interplay in RA is complex, and many cytokines show pleiotropic actions on different cell types. To reduce the complexity and to simplify this interplay, cytokines can roughly be divided into two groups, the proinflammatory and anti-inflammatory mediators. Controlling the balance between these two groups is an essential therapeutic aim in RA.

In view of recent findings highlighting the existence of a gut-joint axis [3], we investigated the impact of a short-term high-fiber dietary intervention on this balance. Soluble fibers can be degraded into short-chain fatty acids (SCFA), such as acetate, propionate and butyrate, by gut microbial fermentation processes. Next to their important role as an energy source for intestinal epithelial cells, SCFA have been recognized as anti-inflammatory mediators involved in systemic immune functions [4]. Besides the described direct effects of SCFA in inducing regulatory T cell numbers [5,6] or inhibiting T cell proliferation, there are numerous reports showing their indirect effects on chemokine or cytokine secretion by macrophages, neutrophils and lymphocytes [4]. Here, we report that short-term high-fiber dietary intervention in RA patients not only increased SCFA but also decreased the known proinflammatory chemokine MCP-1 (CCL2) and the proinflammatory cytokines IL-18 and IL-33 [7,8,9]. These findings will help to further improve existing pharmacological therapies in RA patients by applying dietary interventions.

## 2. Materials and Methods

### 2.1. Study Subjects

36 patients fulfilling the RA classification criteria of the American College of Rheumatology/European League Against Rheumatism (ACR/EULAR) and being in a clinical remission state (Disease Activity Score (DAS) ≤ 2.6) were recruited for this prospective study at the outpatient clinic of the Medical department 3 of the University Clinic Erlangen, Germany. The study was conducted from November 2018 to January 2019. All participants were treated as consecutive cases after receiving their informed written consent. The study participants were on stable treatment using disease-modifying antirheumatic drugs (DMARDs) (conventional synthetic, targeted synthetic, or biological DMARDs CMARD) and got detailed instructions on the additional use of the high-fiber dietary supplement. All study subjects continued their established standard medication throughout the prospective study, and compliance was confirmed on a weekly basis during the distribution of new high-fiber cereal. The habitual fiber intake of the patients was determined using an altered version of the food frequency questionnaire (FFQ) developed for use in the German Health Examination (DEGS) Survey for Adults, without a significant change (*p* = 0.5416) within the 28-day study period. Stool and blood samples of all study participants were collected at baseline and 28 days. Systemic SCFA levels, microbiota composition and inflammation-associated cytokines in the serum were analyzed at baseline and 28 days. The study was approved by the Ethics Committee of Medical University Erlangen-Nürnberg (approval number 357-17B).

### 2.2. Intervention

Bakery products account for around 70% of all consumed cereal products. To introduce SCFA in commonly consumed foods, bars and cereal containing 50% dietary fibers and a wide range of prebiotic ingredients to support maximum SCFA production were produced. In agreement with the FDA rules (21 CFR 101.9(c)(6)(i)) for “dietary fiber”, nondigestible soluble and insoluble carbohydrates (with three or more monomeric units) were added to ensure physiological effects beneficial to human health. An initial in-men study including healthy volunteers was performed to select a subsequent dietary fiber composition that was highly effective for improving SCFA production in humans: oat flakes, psyllium husk, ground flaxseed, inulin, guar gum, coconut, arrowroot flour and hemp flour. As a nonsynthetic preservative, 0.5% cinnamon was added, thereby also resulting in a generally accepted flavor. A certified bakery mixed the ingredients according to standard processes. Baked bars and cereal were prepackaged into 30-g portions in vacuum packaging to guarantee a longer shelf life of the dietary supplement.

### 2.3. Assessment of Safety (Side Effects, Accompanying Medication and Tolerability)

Side effects and accompanying medication were recorded throughout the study period. The assessment of tolerability was performed at the end of the study. The overall tolerability was categorized as “well tolerated”, “slightly unpleasant” or “very unpleasant”.

### 2.4. Data Analysis and Statistics

Appropriate descriptive statistics were used to tabulate and summarize the demographic and clinical characteristics of patients. The primary end-point of the present prospective study in patients with RA was to assess the impact of short-term high-fiber dietary supplementation on the systemic SCFA levels, changes in gut microbiota composition, and systemic levels of arthritis-associated inflammatory cytokines.

### 2.5. SCFA Measurements

50 µL of serum was transferred into a 2-mL reaction tube. The whole extraction process was performed on ice. 5 µL of 33% HCl was added to each sample, followed by vigorous mixing for one minute. After adding one milliliter of diethyl ether, samples were vortexed for one min and centrifuged for 3 min at 4 °C. The upper organic layer was transferred into two-milliliter gas chromatography (GC) vials. 100 µL of each SCFA calibration standard (Sigma, St. Louis, MO, USA) was diluted in water to concentrations of 0, 0.5, 1, 5 and 10 mM for the calibration process and extracted with the same process as the samples. 1 µL of each sample was injected with a split ratio of 20:1 on a Famewax, 20 m × 0.25 mm iD, 0.25 µm df capillary column (Restek, Bad Homburg, Germany) for a GC mass spectrometric (GCMS) analysis. The GC was coupled with an AOC20S autosampler and an AOC20i auto injector (Shimadzu, Kyoto, Japan). Through standard acquisition and by means of the NIST08 MS library, an injection temperature of 240 °C at a rate of 9 °C/min was defined. In a range of 25–150 *m*/*z*, the full-scan MS were recorded (0.5 s/scan). For the quantification, the extracted ion chromatogram peaks were integrated for the following ion species: *m*/*z* 45 for acetate eluted at 7.8 min, *m*/*z* 74 for propionate eluted at 9.6 min, and *m*/*z* 60 for butyrate eluted at 11.5 min. Data processing was performed by using the GCMSsolution software (Shimadzu, Kyoto, Japan).

### 2.6. Assessment of Human Cytokine Levels in the Serum

Human cytokine levels were analyzed using the LEGENDplex^TM^ human Inflammation Panel 1 (Biolegend, San Diego, CA, USA). A 96-well V-bottom plate was loaded with duplicates, with 25 µL for each sample or standard. 25 µL of mixed beads and 25 µL of assay buffer were added to each sample well. To each standard well, 25 µL of mixed beads and 25 µL of Matrix B were added. The plate was sealed, covered in foil and shaken at 800 rpm for 2 h at room temperature. Then, the plate was centrifuged at 1050 rpm for 5 min, the supernatant was discarded and 25 µL of detection antibody were added to each well. The plate was sealed, covered in foil and shaken at 800 rpm for 1 h at room temperature. 25 µL of Streptavidin-Phycoerythrin (PE) were added directly to each well. The plate was sealed, covered in foil and shaken at 800 rpm for 30 min at room temperature. After centrifugation at 1050 rpm, the supernatant was discarded and 200 µL of wash buffer were added to each well and incubated for 1 min. The plate was centrifuged at 1050 rpm, and the supernatant was discarded and resuspended in 100 µL of 1X wash buffer. The samples were acquired on a Cytoflex S (Beckman Coulter, Brea, CA, USA) and were analyzed with the Legendplex 8.0 Software (Biolegend, San Diego, CA, USA).

### 2.7. 16S rRNA Gene Sequencing-Based Microbiome Analysis

To extract genomic DNA from stool samples, the Qiamp Fast DNA Stool extraction kit (Qiagen, Venlo, Netherlands) was used, following the manufacturer’s instruction. The NEBNext Q5 Host Start Hifi PCR Master Mix (NEB) was utilized for amplification of the V3–V4 region of the bacterial 16S rRNA gene. The purification of amplified fragments was done with AMPure XP Beads (Beckmann Coulter Genomics, Brea, California). This was combined and analyzed by a “2 × 300 bp paired-end” sequencing on a Illumina MiSeq machine. The Usearch10 MicrobiomeAnalyst package [10] was used to perform a quality control, OTU table generation and bioinformatics analysis [11]. Classification was done by utilizing the “Ribosomal database project” (RDP release 16).

### 2.8. Statistical Analysis

All data are presented as mean ± SD unless otherwise stated, in the figure legends. All relevant data are available from the authors. Analysis was performed using Student’s *t*-test. *p*-values of 0.05 were considered significant and are shown as *p* < 0.05 (*), *p* < 0.01 (**) or *p* < 0.001 (***). Graph generation and statistical analysis was performed using the GraphPad Prism version 8 (GraphPad, La Jolla, CA, USA).

### 2.9. Data Availability

All relevant data are available from the authors upon reasonable request. 16S rRNA sequencing data are publicly accessible at the European Nucleotide Archive and available under the following accession code: PRJEB40439.

## 3. Results

### 3.1. Patient and Healthy Control Characteristics

Demographic data of participating healthy control subjects are shown in Table 1. Demographic data and disease-specific data of the RA patients are summarized in Table 2.

### 3.2. Effects on SCFA Concentrations in Healthy Controls

**Figure 1:**
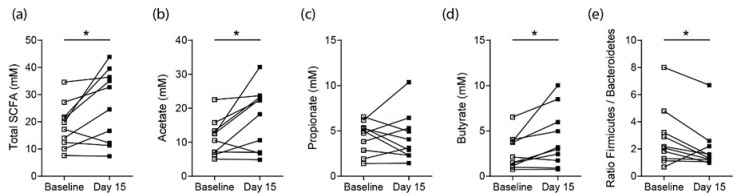
Short-term 15-day high-fiber diet supplementation in healthy controls (*n* = 10) increases SCFA levels. (**a**) Total SCFA levels in stool samples at baseline and day 15 following high-fiber supplementation; Individual (**b**) acetate, (**c**) propionate and (**d**) butyrate stool concentrations of healthy controls; (**e**) Gut microbiota Firmicutes-to-Bacteroidetes ratio based on 16S rRNA sequencing results. Statistical difference was determined by a paired Students *t*-test (Wilcoxon matched-pairs signed rank test). * = *p* < 0.05; ** = *p* < 0.01; *** = *p* < 0.001.

To determine whether short-term high-fiber dietary interventions using one daily fiber bar of 30 g containing 50% of dietary fiber was sufficient to increase SCFA concentrations, stool samples from healthy controls were analyzed by gas chromatography mass spectrometric (GCMS) analysis. For all study participants, the average fiber intake without supplementation was determined using the official questionnaire DEGS from the Robert Koch Institute (Berlin, Germany) at baseline (average = 15.8 g/day) and did not change significantly throughout the study. After 15 days of daily fiber bar consumption, stool samples showed significantly increased total SCFA concentrations (Figure 1a). This total increase was predominantly driven by an increase in acetate and butyrate concentrations, whereas propionate levels remained unchanged (Figure 1b–d). Hence, daily high-fiber bar consumption appears to increase local SCFA concentrations already after 15 days. Moreover, the 16S rRNA analysis of stool samples did not show significant changes in individual bacterial strains (Supplementary Materials Figure S1); however, it revealed a shift in the the Firmicutes-to-Bacteroidetes ratio towards the Bacteroidetes phylum (Figure 1e).

### 3.3. Effects on SCFA Concentrations in RA Patients

**Figure 2:**
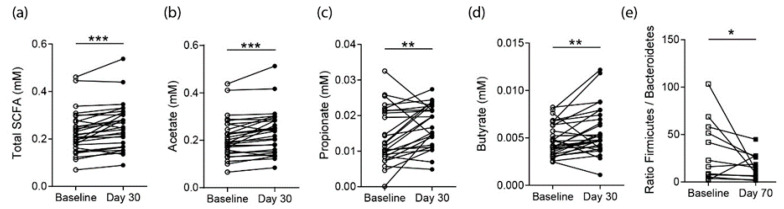
Short-term 30-day high-fiber diet supplementation in RA patients (*n* = 29) increases SCFA levels. (**a**) Total SCFA levels in serum samples at baseline and day 30 following high-fiber supplementation; Individual (**b**) acetate, (**c**) propionate and (**d**) butyrate serum concentrations of RA patients; (**e**) Gut microbiota Firmicutes-to-Bacteroidetes ratio based on 16S rRNA sequencing results (*n* = 15). Statistical difference was determined by a paired Students *t*-test (Wilcoxon matched-pairs signed rank test). * = *p* < 0.05; ** = *p* < 0.01; *** = *p* < 0.001.

Following this observation in healthy controls, we analyzed serum samples from RA patients that did not have a substantial inflammatory activity at baseline due to stable effective DMARD therapy (Table 2). Similar to healthy controls, high-fiber diet interventions by ingestion of one fiber bar daily effectively increased the systemic total SCFA levels measured in the sera of RA patients at day 30 (Figure 2a). Contrary to healthy controls, in RA patients this increase in total SCFA was driven by an increase in acetate, propionate and butyrate together (Figure 2b–d). Following the 30-day fiber intervention phase, there was no significant change in the gut microbiota composition on the genus level (Figure S2). More strikingly we observed a change in the Bacteroidetes-to-Firmicutes ratio towards the Bacteroidetes phylum at day 70, 40 days after the fiber bar intake stopped (Figure 2e).

### 3.4. Effects on Proarthritogenic Cytokine Levels in RA Patients

**Figure 3:**
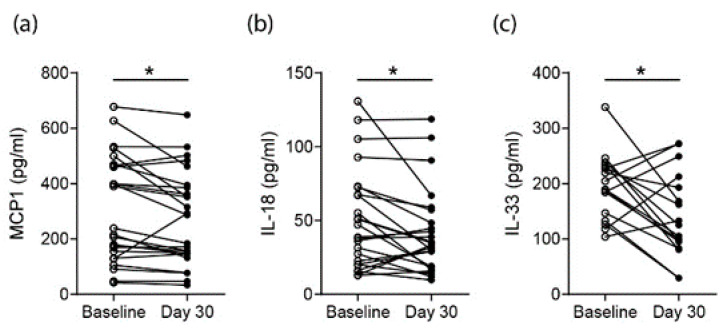
Decreased proinflammatory cytokines following long-term 30-day high-fiber diet supplementation in RA patients (*n* = 29). (**a**) MCP-1 (CCL2), (**b**) IL-18 and (**c**) IL-33 serum concentrations in RA patients at baseline and day 30 following high-fiber supplementation. Statistical difference was determined by a paired Students *t*-test (Wilcoxon matched-pairs signed rank test). *= *p* < 0.05; ** = *p* < 0.01; *** = *p* < 0.001.

Next, we wanted to investigate if the observed increase in SCFA levels affected serum cytokine levels in RA patients. The LEGENDplex panel for human inflammatory cytokines was used to compare baseline vs. day-30 serum cytokine concentrations. The serum concentrations of IL-1β, IFN-a2, IFNγ, TNFα, IL-6, CXCL8 (IL-8), IL-10, IL-12p70, IL-17A and IL-23 did not show any significant differences. However, CCL2 (MCP1), IL-18 and IL-33 were significantly decreased in RA patients following 30 days of high fiber supplementation (Figure 3a–c). This is of interest as these cytokines were described as having arthritogenic properties.

## 4. Discussion

The gut microbiota affects human health and disease via its ability to produce harmful metabolites associated with impaired intestinal barrier function and the development of disease on the one hand or beneficial metabolites that are anti-inflammatory and sustain intestinal barrier function on the other hand. Because diet is a main determinant of the gut microbiota composition, the production of harmful or beneficial microbial metabolites depends, in turn, on dietary intake. De Filippo et al. demonstrated the association of a high dietary fiber intake with increased beneficial microbial metabolites, the short chain fatty acids (SCFA), in children from a rural African village of Burkina Faso by comparing the SCFA levels to those of European children [12]. Diet in Burkina Faso is rich in fibers and predominantly involves whole grains, resistant starch and oligosaccharides that are fermented in the gut by the microbiota, thereby producing SCFA. As a consequence, SCFA levels were increased in children from Burkina Faso when compared to European children from urban parts of Florence, Italy. Other studies revealed that systemic SCFA levels positively correlated with the consumption of fruits, vegetables and legumes and that individuals following a vegan or vegetarian diet, or those on a Mediterranean diet, had increased SCFA concentrations [13].

Although it has become quite clear that a healthy diet has a strong impact on disease prevention [14], there are accumulating reports highlighting the difficulties of patients to follow strict nutritional advice [15,16]. Therefore, we investigated the effect of a high-fiber nutritional intervention on SCFA levels in healthy individuals and chronic RA patients when consuming one single high-fiber bar per day to circumvent the need for further nutritional changes. We show that the short-term (15–30 days) daily consumption of one high-fiber bar significantly increased the beneficial microbial metabolites SCFA, along with a durable change in the Firmicutes-to-Bacteroidetes ratio [17], up to 40 days after the dietary intervention stopped. This is of relevance, as it was shown that SCFAs affected the immune system during inflammatory diseases by modifying gene expression profiles [18,19], cell chemotaxis [20,21], differentiation [6,22,23], proliferation [24,25,26] and apoptosis [27,28]. In addition, SCFAs inhibit histone deacetylases (HDACs) and stimulate histone acetyltransferase [6,29,30,31]. The different mechanisms of SCFA acting on inflammation were recently comprehensively reviewed by Vinolo et al. [4]. Furthermore, we and others could recently also expand the beneficial effect of SCFA to inflammatory arthritis and bone health, both in preclinical models [23,32,33,34,35] and in a first feasibility study in RA patients [36]. The Firmicutes/Bacteroidetes ratio has been shown to be associated with different diseases. It is, for instance, increased in patients with obesity [37] and is associated with a state of chronic inflammation. These factors and the RA-associated dysbiosis impact the Firmicutes/Bacteroidetes ratio and can explain the high variation between healthy controls and the RA patient cohort that we observed here (Control BMI: 22.81 ± 1.66 vs. RA BMI: 26.63 ± 6.4).

Next to the increase in SCFA, we also identified decreased proinflammatory and proarthritogenic chemokine and cytokine levels for monocyte chemoattractant protein-1 (MCP-1/CCL2), IL-18 and IL-33 following a short-term nutritional high-fiber intervention. MCP-1 regulates the migration and infiltration of monocytes/macrophages. The migration of monocytes from the bloodstream across the vascular endothelium is required in response to inflammation. In line with this, both MCP-1 and its receptor CCR2 have been demonstrated to be induced and involved in various diseases [8,38]. This is of interest, as it was shown that MCP-1 concentrations increased in the synovial fluids of patients with RA as compared with osteoarthritis (OA) or other inflammatory and noninflammatory forms of arthritis [39,40]. Patients with RA had increased levels of MCP-1 in their serum as compared to normal volunteers [41]. Another study showed that serum MCP-1 concentrations in RA patients reflected their disease activity [42] and that treatment with a MCP-1 antagonist prevented arthritis in a preclinical mouse model [43].

Besides the direct MCP-1 downregulation by SCFA [44,45], we also observed decreased IL-18 serum levels following a high-fiber intervention in RA patients. Like many other cytokines, IL-18, as a member of the IL-1 superfamily, is pleiotropic, acting in both acquired and innate immunity. In addition to its costimulatory functions on Th1 cytokines, IL-18 acts directly as a proinflammatory cytokine [46]. The level of IL-18 reportedly increased in both the serum and synovial fluid samples of RA patients [39,47,48,49], and IL-18-deficient mice were shown to have a reduced incidence and severity of arthritis in preclinical models [50,51]. IL-33, another proinflammatory cytokine that was shown to exacerbate inflammation in preclinical arthritis models [9,52,53] but that, at the same time, harbors direct effects on bone homeostasis [54,55], was significantly reduced in RA patients following short-term high-fiber dietary interventions.

In summary, the increased levels of SCFA in RA patients following short-term high-fiber dietary interventions correlated with an observed decrease in proinflammatory chemokine MCP-1 and cytokines IL-18 and IL-33 [7,8,9]. All of these affected cytokines had been extensively described for their proarthritogenic effects. This decrease, together with the described anti-inflammatory and antiarthritic effects of SCFA, highlights the potential for high-fiber dietary interventions to complement future therapeutic strategies in RA patients.

## Figures and Tables

**Table 1:** Demographic data and current treatment of participating healthy controls.

Demographic Characteristics (*N* = 10)		
Age, years (mean ± SD)	28 ± 4.87	
BMI, units (mean ± SD)	22.81 ± 1.66	
Females, *N*	0	0%
Ever smoker, *N*	2	20%

BMI: Body Mass Index.

**Table 2:** Patient data and current treatment of study subjects.

**Patient characteristics (*N* = 36)**		
Age, years (mean ± SD)	56.19 ± 7.7	
BMI, units (mean ± SD)	26.63 ± 6.4	
Females, *N*	20	64.51%
Ever smoker, *N*	6	19.35%
**RA-specific data (*N* = 36)**		
Time since disease onset, years (mean ± SD)	11.64 ± 9.39	
Disease activity score (DAS) 28, units (mean ± SD)	2.54 ± 0.28	
Anti-CCP-IgG antibody positive, *N*	15	48.38%
Rheumatoid Factor IgM positive, *N*	12	38.7%
**Accompanying treatment (*N* = 36)**		
Biological disease-modifying anti-rheumatic drugs, N	23	74.10%
Abatacept, *N*	3	9.67%
Rituximab, *N*	7	22.58%
Tocilizumab, *N*	8	25.8%
Tumor Necrosis Factor Inhibitors, N	5	16.12%
Glucocorticoids, *N*	5	16.12%
JAK-inhibitors, *N*	4	12.9%
Methotrexate, *N*	16	51.61%
Other conventional synthetic disease-modifying anti-rheumatic drugs, *N*	1	3.22%

RA: Rheumatoid Arthritis, CCP: Cyclic Citrullinated Peptide, JAK: Janus Kinase.

## Supplementary Materials

The following are available online at https://www.mdpi.com/2072-6643/12/10/3207/s1, Figure S1: 16S rRNA sequencing results from healthy controls, Figure S2: 16S rRNA sequencing results from RA patients.
