# Supplementary material for: Dietary Short-Term Fiber Interventions in Arthritis Patients Increase Systemic SCFA Levels and Regulate Inflammation
Source: Nutrients. 2020 Oct 20;12(10):3207. doi: 10.3390/nu12103207 (PMC7589100; doi:10.3390/nu12103207)
Supplement: Supplementary file 1 [file nutrients-12-03207-s001.pdf]

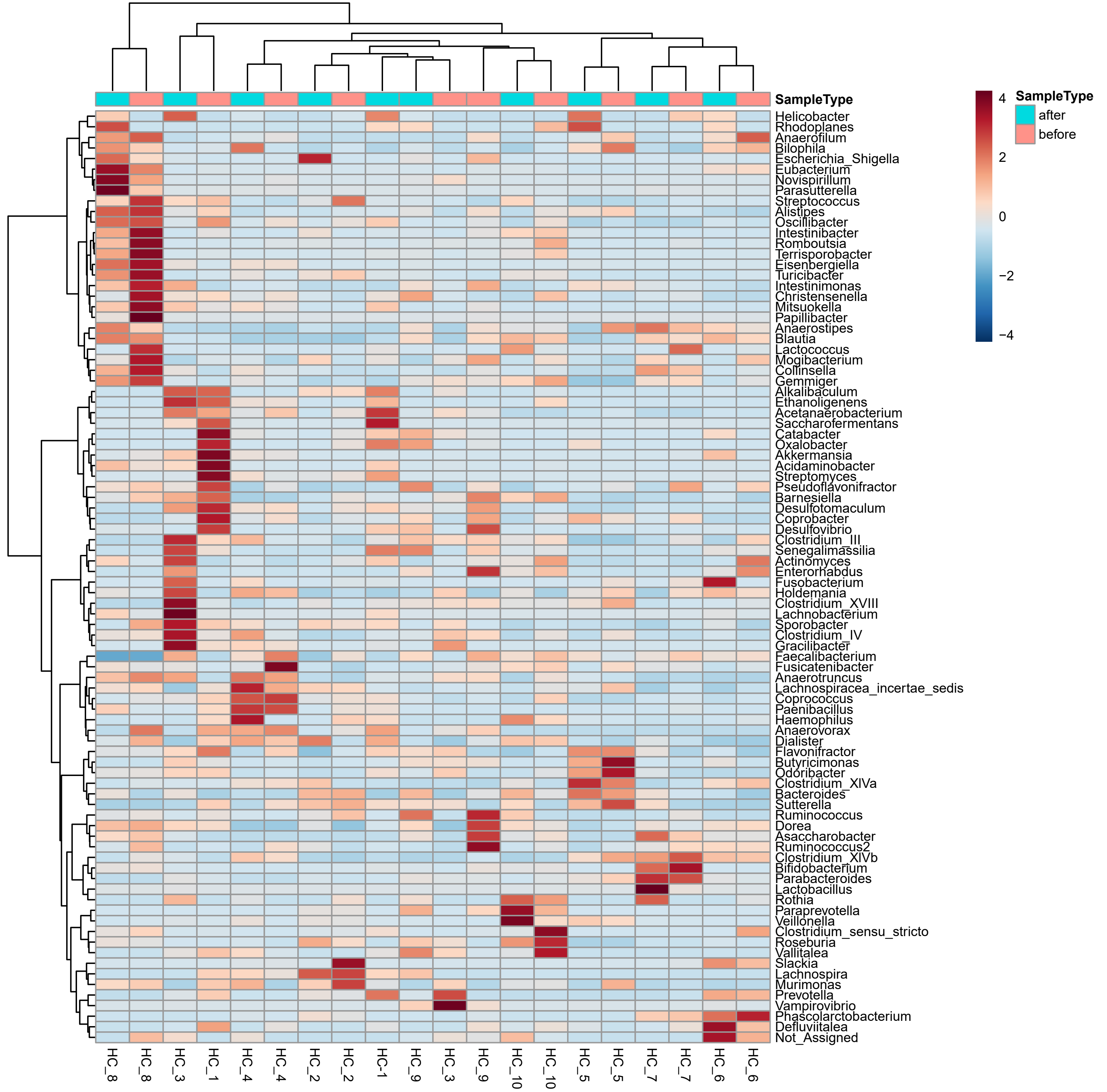

Supplemental Figure 1: 16S rRNA sequencing results from healthy controls (HC) (n=10) before (Day 0) and after (Day 28) high-fiber diet interventions shown as heatmap for the genus levels.

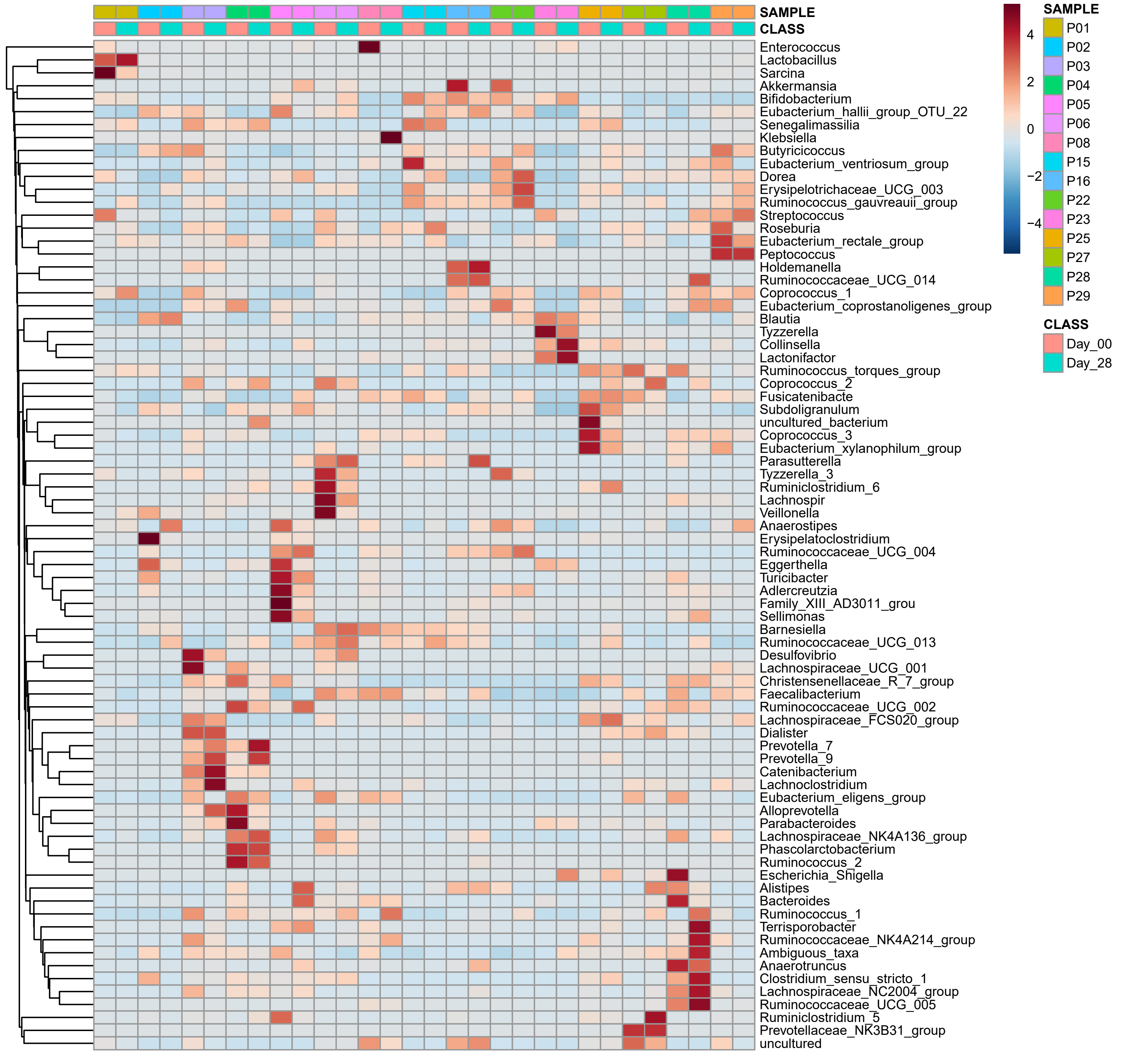

Supplemental Figure 2. 16S rRNA sequencing results from RA patients (n=15) before (Day 0) and after (Day 28) high-fiber diet interventions shown as heatmap on the genus levels.
